# Practice of the new supervised machine learning predictive analytics for glioma patient survival after tumor resection: Experiences in a high-volume Chinese center

**DOI:** 10.3389/fsurg.2022.975022

**Published:** 2023-02-17

**Authors:** Yushan Li, Maodong Ye, Baolong Jia, Linwei Chen, Zubang Zhou

**Affiliations:** ^1^ Department of Ultrasound, Gansu Provincial Hospital, Lanzhou, China; ^2^ Medical Cosmetic Center, First Affiliated Hospital of Shantou University Medical College, Shantou, China; ^3^ Pingliang Second People's Hospital Neurosurgery Department, Pingliang, China; ^4^ Neurosurgery Unit, The First Affiliated Hospital, Sun Yat-sen University, Guangzhou, China

**Keywords:** glioma, machine learning, Gradient Boosting, survival, tumor resection

## Abstract

**Objective:**

This study aims to assess the effectiveness of the Gradient Boosting (GB) algorithm on glioma prognosis prediction and to explore new predictive models for glioma patient survival after tumor resection.

**Methods:**

A cohort of 776 glioma cases (WHO grades II–IV) between 2010 and 2017 was obtained. Clinical characteristics and biomarker information were reviewed. Subsequently, we constructed the conventional Cox survival model and three different supervised machine learning models, including support vector machine (SVM), random survival forest (RSF), Tree GB, and Component GB. Then, the model performance was compared with each other. At last, we also assessed the feature importance of models.

**Results:**

The concordance indexes of the conventional survival model, SVM, RSF, Tree GB, and Component GB were 0.755, 0.787, 0.830, 0.837, and 0.840, respectively. All areas under the cumulative receiver operating characteristic curve of both GB models were above 0.800 at different survival times. Their calibration curves showed good calibration of survival prediction. Meanwhile, the analysis of feature importance revealed Karnofsky performance status, age, tumor subtype, extent of resection, and so on as crucial predictive factors.

**Conclusion:**

Gradient Boosting models performed better in predicting glioma patient survival after tumor resection than other models.

## Introduction

Glioma is the most widely recognized primary tumor in the central nervous system (CNS) (1). Accounting for around 80% of malignant CNS tumors (1), gliomas are composed of lower-grade gliomas [LGGs; World Health Organization (WHO) grades II and III] and grade IV gliomas (glioblastoma, GBM). The treatment of glioma is troublesome, and tumor resection is the main approach to treatment. Due to the large heterogeneity between different kinds of gliomas, the prognosis of glioma patients is diverse, and the survival always ranges from a few months to 10 years (2, 3). Obviously, GBM was supposed to have a poorer prognosis than diffuse low-grade and intermediate-grade gliomas for its characteristics of invading growth and easy recurrence. However, along with the presence of certain molecular markers and various clinical characteristics, including age, Karnofsky performance status (KPS), symptoms, and so on, the prognosis varies even in this most malignant type of glioma, GBM. Predicting glioma patient survival after tumor resection still remains a great challenge for clinical doctors.

Nowadays, there have been endeavors, mainly in three directions, to explore useful predictive models for glioma prognosis. Some researchers have focused on traditional multivariate Cox regression models with several certain prognostic factors. For example, Gittleman et al. (4) developed a survival nomogram for LGGs with independent validation. Meanwhile, some turn to new biomarkers for the construction of models. Not long ago, Zhang et al. (5) constructed a novel model using immune-related gene signature, which is also effective in predicting overall survival in primary LGG. What is more, some researchers have concentrated on radiomics feature prediction models and made some achievements (6). Albeit the effort in putting forward these models, some shortcomings limit their usefulness and availability of these models. First, the traditional statistic approach has a huge limitation: its analysis is based on the condition of a linear relationship and might miss the nonlinear relationship between input and outcome. In other words, this approach cannot fully use medical information, which makes it unable to adjust to the era of big data. Second, as Jakola et al. (7) claimed, a pure biomarker approach for prediction, such as gene signature model, is of limited value because tumor classes and tumor cells are neither stable over time nor homogeneous throughout the lesion tissue. Third, prediction models based on radiomics features are powerful and promising, but we acknowledge that the techniques are at an early stage and available only at a limited number of centers and not readily validated in medical practice yet (7). Therefore, it is still necessary to explore a new predictive model based on the algorithm suited to the big-data era, with the combination of common clinical features and reliable biomarkers as prognostic factors.

Recently, supervised machine learning (ML) methods have demonstrated precise predictive capacity, being progressively utilized in the prognosis prediction of different diseases (8). The supervised ML approach is a kind of data-driven analysis method, including support vector machine (SVM) (9), decision tree (10), and so on, which integrates multiple risk factors into a predictive algorithm and performs well with complex information (11). Gradient Boosting (GB) is one of the supervised ML algorithms. Although it was strange for medical workers, this ML algorithm did have a good performance in medical scenes, such as predicting the survival outcome of triple-negative breast cancer (12) and the recurrence of colorectal cancer (13). So far, studies seldom used Gradient Boosting to analyze and predict glioma prognosis. This study was conducted to assess its effectiveness on glioma prognosis prediction and to explore new predictive models for glioma patient survival after tumor resection.

## Patients and methods

### Patients

Approved by the Institutional Review Board of Sun Yat-sen University, this study was conducted in the Neurosurgery unit, the First Affiliated Hospital, Sun Yat-sen University, a high-volume central center that performs approximately 100 glioma surgeries yearly. In accordance with the guidelines for retrospective study in our institution, the institutional review board waived the requirement for patients' informed consent. Our study only included cases of astrocytoma, oligodendroglioma and oligoastrocytoma, anaplastic astrocytoma, oligodendroglioma and oligoastrocytoma, and glioblastoma. A cohort of 776 glioma cases (WHO grades II–IV) between 2010 and 2017 was obtained. This consecutive malignant series consisted of 74 cases of WHO grade III (anaplastic astrocytoma, oligodendroglioma, and oligoastrocytoma), 268 cases of WHO grade IV (glioblastoma), and 434 cases of WHO grade II (astrocytoma, oligodendroglioma, and oligoastrocytoma).

### Clinical characteristics

Most data were accessible through the hospital database. All data were extracted into two copies of a standardized form by two research assistants independently and integrated into the final file version by a third. Discrepancies were discussed and resolved by consensus. The extracted characteristics include age at surgery, gender, symptoms (seizures, headaches/dizziness, nausea/vomiting, limb dysfunction, blurred vision, or other cranial nerve deficit), duration of the first presenting symptom, preoperative KPS, tumor size and location, time of surgery, extent of resection (gross-total resection and others), tumor subtype, treatment after surgery (chemotherapy and/or radiotherapy), survival status (alive or dead), and survival/follow-up time. The subtype of glioma was reviewed by a pathologist according to the latest 2016 WHO criteria (14). The deficit of motor, visual, or cranial never function was confirmed by the proof of physical examination, diffusion tensor imaging (DTI)-based tractography, and so on. The same as the definition by Okamoto et al. (15), the extent of resection was categorized, where gross-total resection was defined as residual tumor less than 5%. The follow-up data were collected until December 2019. Survival/follow-up time was calculated from the date of tumor resection to death (any cause) or censor (still survived) in December 2019. All patients were followed up at the regular interval of 3 months for the initial 3 years and afterward followed every 1 year until death. The last follow-up for every single accessible patient was finished in December 2019.

### Biomarkers

Biomarkers’ detection, including immunohistochemistry (IHC) and molecular genetics, was performed on histological specimens that were obtained at the time of resection surgery prior to chemotherapy and/or radiotherapy treatment. The detection of kit67, p53, vimentin, and glial fibrillary acidic protein (GFAP) was performed using immunohistochemical stains in glioma by standard techniques that were described previously (16). For the specimen with p53 immunohistochemical stain, the presence of strong positive tumor nuclei in more than 10% of cells was marked as immunopositive, which indicated the mutational status of TP53 (17). The immunopositivity of vimentin was identified when more than 25% of tumor nuclei stained positive with vimentin IHC stain. GFAP immunopositivity was marked when any tumor nuclei were positive with GFAP IHC stain. The Ki-67 index was recorded as the average percentage of the positive ones on the total number of nuclei at 400× magnification, where “≥10%” represented high Ki-67 expression (18). As for molecular genetics, the biomarker we detected was methylation of the O^6^-methylgaunine-DNA-methyltransferase (MGMT) promoter. This test was done using methylation-specific PCR.

### Supervised machine learning algorithm

SVM, as a machine learning algorithm, has been widely used in the prognosis of diseases. Decision tree is a well-known ML approach for statistical problems, which represents the mapping relationship between properties and values. It consists of a root node, internal nodes, and leaf nodes, where leaf nodes correspond to values represented by the path from the root node to the leaf node. Decision tree can be used for survival analysis (19). Here, we used survival decision tree as the base learner of random forests (RFs). RF is an ensemble tree method whose final prediction is the average of all predictions from every tree in the forest. RF performs better in prediction than a single tree because a combination of predictions from separate methods could substantially promote prediction performance (20). Random survival forest (RSF) is an adaptation of random forest, which is designed for the analysis of survival data (21).

GB is an ML technique that can be used for survival analysis. Here, we used component-wise least squares and survival decision tree as two types of base learners, respectively. The Gradient Boosting algorithm produces different weak prediction models (for instance, component-wise least squares) at each step and combines them into a total model at different weights. The prediction of the weak model that Gradient Boosting produced at each step generates a unanimous gradient direction of the loss function. The details have been described previously (22).

### Model evaluation

Harrell's concordance index (*c*-index), defined as the ratio of correctly ordered (concordant) pairs to comparable pairs, is a measure of the rank correlation between predicted risk scores and observed time points. A value of 1 refers to perfect prediction, while a value of 0.5 means that prediction does not perform better than random guessing.

The area under the receiver operating characteristic curve (ROC curve) is often used to assess the discrimination of the binary classification model. When extending the ROC curve to survival time, it gives rise to the time-dependent cumulative ROC curve at a certain survival time *t*. The area under the cumulative ROC curve (AUC) at time *t* indicates how well a model can distinguish subjects who will experience an event by time *t* from those who will not.

The calibration curve is a graphical measure of the calibration of the model, which is a linear plot with the predicted event on the *x*-axis and the observed event on the *y*-axis. Good calibration would be matched by a regression line with a 45° slope.

To fully capture the true utility of a prediction model, the sensitivity and specificity of models for predicting 6-, 12-, 36-, and 60-month survival were calculated after determining the optimal threshold through the ROC curve.

### Model construction

All clinical characteristics and biomarker information were included in the model training set as variables. The missing values of variables were filled with multiple imputations. Here, we randomly split the data into a training set and a test set at an 8:2 ratio using the train_test_split function in the scikit-learning module of Python (version 3.7). The scikit-survival module (version 0.12.1) was used to construct ML models, SVM, RF, Tree GB, and Component GB. ML algorithms involve many hyperparameters that are significant for performance prediction. The optimal combination of hyperparameters was determined using the method of grid search. During every cross-validation, 1/3 of the data in the training set were randomly excluded as out-of-bag (OOB) data for validation. For different combinations of hyperparameters, the mean *c*-index on the validation data was calculated after 50 times cross-validation. The hyperparameter combination with the best *c*-index was selected as optimum. After constructing the model, we usually assess the feature importance by calculating its contribution to the *c*-index, namely, the decrease of *c*-index after discombobulating the relationship of this feature with survival.

To compare the performance difference between ML models and conventional survival models, we also built the Cox proportional hazards model. Three continuous variables, age at surgery, preoperative KPS, and tumor size, were transformed into categorical variables to obtain the best model prediction performance. Cutoffs for these variables were 50 years, 70 cm, and 55 cm, respectively. All variables were entered into the model step by step, and the final model only included variables with a significant risk ratio.

### Statistical analysis

Mean ± SD or median (IQR) was chosen to describe continuous variables regarding their statistical distribution, while categorical variables were expressed in the form of example numbers (%). *P* < 0.05 was set as the criteria of statistical significance in all analyses. The confidence interval (CI) of the AUC was computed by the bootstrap method, while 95% CI was computed with 2,000 stratified bootstrap replicates. The comparisons of the *c*-index between different models were conducted using the R package Survcomp.

## Results

### Characteristic overview

The sociodemographic and characteristics of the study population are presented in Table 1. The most frequent symptom was headaches or dizziness, while the most frequent tumor location and subtype were parietal lobe and diffuse astrocytoma, respectively. The medians of the duration of the first presenting symptom, preoperative KPS, and tumor size were 1.90 months, 60 mm, and 45.00 mm, respectively. Gross-total resection was adopted in 90.0% of patients. The immunopositivity of GFAP, Vimentin, and p53 was observed in more than half of the patients. The median of survival time was 32.65 months.

**Table 1:** Clinical characteristics and biomarkers of patients.

** **	**Total (*N* = 776)**
**Demographics**	
**Age at surgery, years, median (IQR)**	38.00 (24.25–53.00)
**Age > 50 years (%)**	29.0% (225/776)
**Sex (M, %)**	58.1% (451/776)
**Symptoms**	
**Seizures (%)**	34.7% (267/769)
**Headaches/dizziness (%)**	61.5% (473/769)
**Nausea/vomiting (%)**	25.0% (192/769)
**Limb dysfunction (%)**	18.7% (144/769)
**Blurred vision (%)**	9.5% (73/769)
**Other cranial nerve deficit (%)**	16.6% (128/769)
**Duration of symptom, m, median (IQR)**	1.90 (0.70–6.00)
**Preoperative KPS**	
KPS, median (IQR)	
**60.00 (50.00–90.00)**	
**KPS > 70 (%)**	**37.9% (275/726)**
**Tumor**	
**Size, mm, median (IQR)**	**45.00 (32.00—60.00)**
**Size > 55 cm (%)**	**32.0% (239/747)**
**Tumor location**	
**Frontal lobe (%)**	**42.4% (327/771)**
**Temporal lobe (%)**	**17.5% (135/771)**
**Parietal lobe (%)**	**32.9% (254/771)**
**Occipital lobe (%)**	**8.3% (64/771)**
**Infra-tentorial (%)**	**10.1% (78/771)**
**Others (%)**	**16.1% (124/771)**
**Extent of resection**	
**Gross-total resection (%)**	**90.0% (691/768)**
**Others (%)**	**10.0% (77/768)**
**Tumor subtype**	
**Diffuse astrocytoma, IDH mutant (%)**	**9.0% (70/776)**
**Diffuse astrocytoma, IDH wildtype (%)**	**0.5% (4/776)**
**Diffuse astrocytoma, NOS (%)**	**30.2% (234/776)**
**Oligodendroglioma, IDH mutant (%)**	**3.1% (24/776)**
**Oligodendroglioma, NOS (%)**	**11.3% (88/776)**
**Oligoastrocytoma, NOS (%)**	**1.8% (14/776)**
**Anaplastic astrocytoma, IDH mutant (%)**	**1.2% (9/776)**
**Anaplastic astrocytoma, IDH wildtype (%)**	**0.1% (1/776)**
**Anaplastic astrocytoma, NOS (%)**	**4.9% (38/776)**
**Anaplastic oligodendroglioma, IDH mutant (%)**	**0.4% (3/776)**
**Anaplastic oligodendroglioma, NOS (%)**	**2.7% (21/776)**
**Anaplastic oligoastrocytoma, NOS (%)**	**0.3% (2/776)**
**Glioblastoma, IDH mutant (%)**	**1.2% (9/776)**
**Glioblastoma, IDH wildtype (%)**	**7.9% (61/776)**
**Glioblastoma, NOS (%)**	**25.5% (198/776)**
**Treatment strategy**	
**Chemotherapy (%)**	**74.8% (564/754)**
**Radiotherapy (%)**	**46.6% (355/761)**
**Biomarkers**	
**Ki-67, median (IQR)**	**0.12 (0.05—0.30)**
**GFAP immunopositivity (%)**	**79.7% (570/715)**
**Vimentin immunopositivity (%)**	**83.6% (532/636)**
**MGMT promoter methylation (%)**	**41.7% (301/722)**
**p53 immunopositivity (%)**	**57.6% (411/713)**
**Follow-up**	
**Status of death (%)**	**71.5% (555/776)**
**Survival time, m, median (IQR)**	**32.65 (13.03–56.23)**

KPS, Karnofsky performance status; GFAP, glial fibrillary acidic protein; MGMT, O^6^-methylgaunine-DNA-methyltransferase.

**Figure 1:**
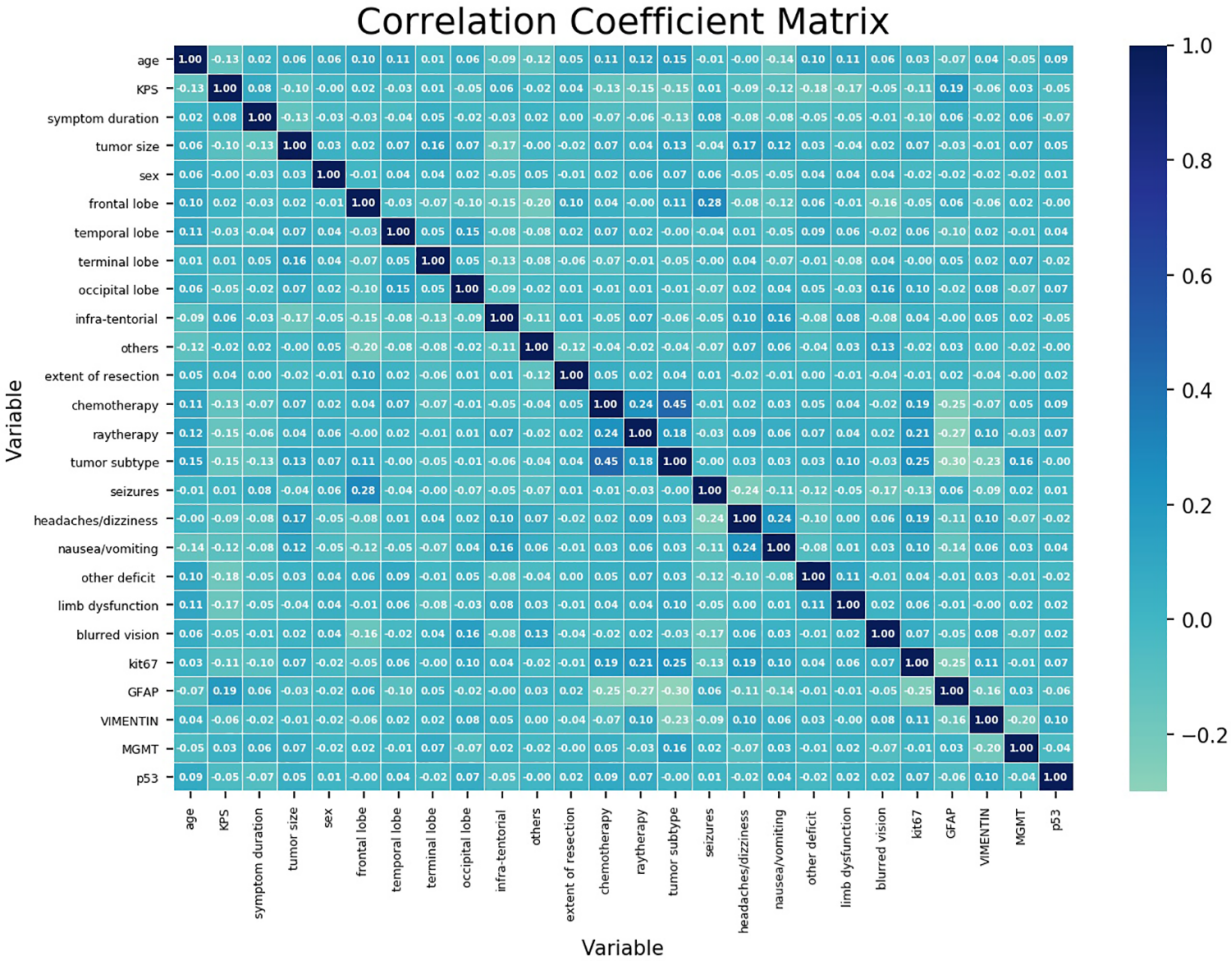
Correlation coefficient matrix of each variable. Each coefficient is annotated. The closer it gets to 1, the more positively correlated it is. The closer it gets to −1, the more negatively correlated it is.

Figure 1 shows the correlation coefficient between each independent variable. It demonstrated low correlation between each variable.

### Model performance

The flow chart of model construction is shown in Supplementary Figure S1. Supplementary Table S1 shows the detailed descriptions of the selected modules, classes, and hyperparameters in Python for each model, including the Cox survival model and supervised ML models.

The five models are compared in Table 2. The Cox proportional hazards model had the worst performance, with a concordance index of 0.755 for the test set. The SVM model was observed to have relatively poor performance, with a *c*-index of 0.787 for the test set. The Tree GB survival model ranked second, with a *c*-index of 0.837, while the RSF model ranked third, with a *c*-index of 0.830. The Component GB survival model had the best prediction performance, with a *c*-index of 0.840. In addition, we also compared the *c*-index values of different models on the test set. The *c*-index values of Tree GB and Component GB survival models were significantly higher than those of the Cox proportional hazards model (*P* < 0.05) and SVM model (*P* < 0.05). Although the comparison results of the RF model were not significant (*P* values were 0.332 and 0.112, respectively), relatively superior performances of Tree GB and Component GB models were still observed. The reason for no statistical significance could be attributed to the little sample size of the test set to a certain extent.

**Table 2:** Concordance indexes of models for the training set and test set.

** **	**Training** s**et**	**Testing** s**et**
**Cox proportional hazards model**	0.819	0.755
**Support vector machine model**	0.838	0.787
**Random survival forest model**	0.875	0.830
**Tree Gradient Boosting survival model**	0.905	0.837
**Component Gradient Boosting survival model**	0.857	0.840

**Figure 2:**
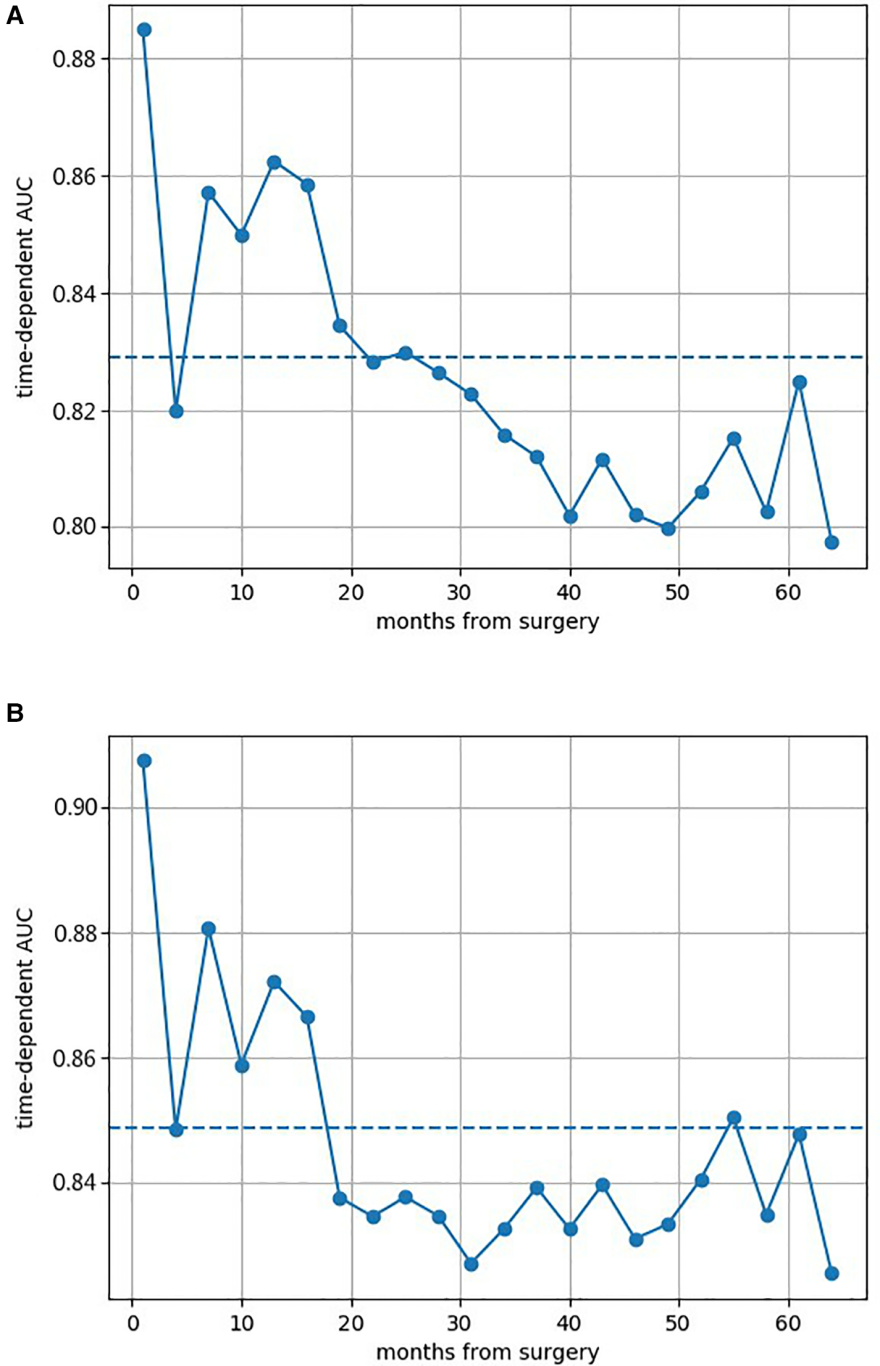
Area under the cumulative ROC curves of both two Gradient Boosting models at different survival times. (**A**) AUC of the Component GB survival model at different survival times. (**B**) AUC of the Tree GB survival model at different survival times. ROC curve, receiver operating characteristic curve; AUC, area under the curve; GB, Gradient Boosting.

Figure 2 shows the AUCs of both GB models. All AUCs at different survival times were above 0.800, which indicated the excellent discrimination of models. The prediction AUC and CI values of both GB models’ for 6-, 12-, 36-, and 60-month survival are specifically listed in Supplementary Tables S2 and S3, which highlighted superior predictive performance. The calibration curves of both GB models are shown in Supplementary Figures S2 and S3, where good calibration was found in survival prediction. Based on the optimal thresholds, the Tree GB model predicted 6-, 12-, 36-, and 60-month survival with 94.4%, 90.6%, 99.3%, and 100% sensitivity and 91.3%, 85.7%, 71.3%, and 73.8% specificity, while the Component GB model predicted the survival results with 90.0%, 73.5%, 85.2%, and 90.4% sensitivity and 84.1%, 92.9%, 87.5%, and 82.8% specificity, respectively. The results are listed in Supplementary Tables S4 and S5.

### Feature importance

From Table 3, we found that KPS, the tumor subtype of glioblastoma not otherwise specified (NOS), age, tumor size, and the tumor subtype of oligodendroglioma (NOS) ranked top five in the Tree GB survival model in terms of the feature importance. As for the Component GB survival model, it was KPS, the tumor subtype of glioblastoma (NOS), age, extent of resection, and tumor size that ranked the top five. As described in Supplementary Figure S4, significant variables included in the final Cox proportional hazards model were KPS, age, tumor size, tumor subtype, extent of resection, chemotherapy, radiotherapy, p53 immunopositivity, and methylation of the MGMT promoter.

**Table 3:** Top 15 feature importance of two Gradient Boosting models.

**Feature importance rank**	**Tree Gradient Boosting model**	**Component Gradient Boosting model**
**1**	KPS	KPS
**2**	Glioblastoma, NOS	Glioblastoma, NOS
**3**	Age	Age
**4**	Tumor size	Extent of resection, others
**5**	Oligodendroglioma, NOS	Tumor size
**6**	Extent of resection, others	Glioblastoma, IDH wildtype
**7**	MGMT promoter methylation negative	MGMT promoter methylation positive
**8**	Glioblastoma, IDH wildtype	Oligodendroglioma, NOS
**9**	Extent of resection, gross-total resection	With radiotherapy
**10**	Oligoastrocytoma, NOS	p53 negative
**11**	MGMT promoter methylation positive	With chemotherapy
**12**	With radiotherapy	Oligoastrocytoma, NOS
**13**	p53 positive	Diffuse astrocytoma, NOS
**14**	With chemotherapy	MGMT promoter methylation negative
**15**	p53 negative	Glioblastoma, IDH mutant

KPS, Karnofsky performance status; MGMT, O^6^-methylgaunine-DNA-methyltransferase.

### Sensitivity analysis

We also performed sensitivity analysis to detect the robustness of ML model prediction performance. Training and testing with variables without imputation, the *c*-indexes of ML survival models are listed in Table 4. All *c*-indexes were at a high level (above 0.800), which indicated the robustness of ML model prediction performance. Considering the large heterogeneity of different types of gliomas, we deliberately tested the prediction performance of ML models on three most common gliomas, namely, diffuse astrocytoma, oligodendroglioma, and glioblastoma. As can be seen from Supplementary Table S6, the *c*-indexes were almost at the level of about 0.8, which proves that the model is compatible with different types of gliomas.

**Table 4:** Concordance indexes of models for variables without imputation.

**Variable without imputation**	**Tree Gradient Boosting model**	**Component Gradient Boosting model**
**Symptoms**	0.851	0.857
**Duration of symptom**	0.831	0.837
**Preoperative KPS**	0.810	0.821
**Tumor size**	0.834	0.844
**Tumor location**	0.829	0.857
**Extent of resection**	0.803	0.826
**Chemotherapy**	0.835	0.836
**Radiotherapy**	0.842	0.852
**Ki-67**	0.820	0.835
**GFAP immunopositivity**	0.829	0.840
**Vimentin immunopositivity**	0.823	0.846
**MGMT promoter methylation**	0.845	0.859
**p53 immunopositivity**	0.835	0.837

KPS, Karnofsky performance status; GFAP, glial fibrillary acidic protein; MGMT, O^6^-methylgaunine-DNA-methyltransferase.

## Discussion

This study was designed to assess the effectiveness of the supervised ML algorithm, especially Gradient Boosting, in the prediction of glioma patient survival after tumor resection and to explore new predictive models useful for medical workers. Judging by Harrell's concordance index of the training set and test set, the Gradient Boosting algorithm ranked first on prediction performance. There were differences in prediction performance between Tree GB and Component GB algorithms. Tree GB showed better performance on the training set (*c*-index: 0.905) but worst performance on the test set (*c*-index: 0.837) than Component GB, which implied a trend of overfitting. By the way, considering the discrimination and calibration through time-dependent cumulative ROC curves and sensitivity, specificity, and calibration curves, Tree and Component GB were both good at predicting 6-, 12-, 36-, and 60-month survival after surgery. The results of the sensitive analysis revealed that both GB models were stable at the prediction outcome.

At the same time, the feature importance of ML models was also assessed. The top 15 important features in Tree or Component GB models could be reduced to nine variables, namely, KPS, age, tumor size, tumor subtype, extent of resection, chemotherapy, radiotherapy, p53 immunopositivity, and methylation of the MGMT promoter. It was in line with the significant variables included in the Cox proportional hazards model.

Consistent with the results of previous studies (23, 24), our result revealed that KPS influenced glioma patient survival after resection surgery. KPS or similar crude scales are commonly seen methods to evaluate gross functional status and have been repeatedly described as prognostic factors in the management of glioma patients (23, 24). Also, age is one of the most established prognostic factors in patients with malignant gliomas, regardless of lower-grade (24, 25) or higher-grade gliomas (26). As claimed by Paugh et al. (27), the substantial differences in the molecular features underlying age-stratified gliomas might lead to different treatment responses, accounting for different survival outcomes. A cutoff value of 55 years has been reported repeatedly to stratify glioma patients, while significantly impaired survival is always observed in those 55 years and above. Here, our study confirmed advanced age as an unfavorable prognostic factor once more. Previous studies (28, 29) have shown a strong association between preoperative tumor size and glioma survival, which is in line with the finding of our research. Regarding the extent of resection, complete curative resection is thought impossible due to the lack of clear tumor borders and the invasive behavior of the tumor. Although a number of studies (30, 31) have demonstrated that maximal resection substantially improves progression-free and overall survival, it has also been reported that aggressive glioma resection might increase the risk of postoperative complications and lead to worse survival prognosis. Therefore, the relationship between the extent of surgical resection and patient outcome still remains controversial. Even so, our results showed a positive correlation between gross-total resection and prognosis improvement for patients compared to partial resection or biopsy. In view of chemotherapy and radiotherapy, they are crucial elements in the treatment plan of glioma patients. Postoperative adjuvant radiotherapy and chemotherapy have always been recommended to start within 2–4 weeks after surgical resection and have proven to be significant prognostic factors by previous studies (32, 33) and this study. Tumor subtype is one the most commonly recognized prognostic factors, and the subtype based on the latest 2016 WHO criteria helps to predict patient prognosis more accurately. Here, our research also served as evidence of the critical role of tumor subtype in glioma management.

Then, it comes to biomarker information. MGMT is a DNA repair protein that removes alkyl groups and adducts at the O^6^ position of guanine, protecting the cell against mutagenic effects. Promoter methylation of MGMT causes silencing of the MGMT gene and loss of protein expression, accounting for the accumulation of DNA damage and increased sensitivity to temozolomide-based chemoradiotherapy. A prognostic effect of MGMT promoter methylation in patients with lower-grade (34) or higher-grade (35) glioma has already been observed. Located on human chromosome 17p13, the p53 gene is a tumor suppressor and has been detected to regulate apoptosis, inhibit DNA replication, and control cell motility and invasion. As a consequence of p53 gene mutation, the mutant p53 protein escapes from degradation and accumulates in the cells, leading to positive staining by IHC. A meta-analysis concluded that p53 immunopositivity has effective usefulness in analyzing the prognosis of glioma patients (36). As for GFAP, Vimentin, and Ki-67, there exist a number of research studies (37–39) concentrating on their prognostic value. However, our analysis only validated the essential prognostic value of MGMT promoter methylation and p53 immunopositivity, and the other biomarkers need to be further evaluated.

There were several limitations. First, this was a single-center study, which might make the analysis potentially prone to bias and limit the generalization of supervised ML models. Second, the study included cases that occurred before 2016, where the glioma subtype classification at that time was different from the recent 2016 WHO criteria, causing half of those cases to lack evidence of subdivision (for instance, isocitrate dehydrogenase (IDH)) for the latter classification criteria. This might influence the calibration and discrimination of prediction models. Nevertheless, we believed our research has merit, given it is the first study to apply Gradient Boosting algorithms to glioma prognosis prediction. We had constructed predictive models successfully and also found that Gradient Boosting models were more likely to improve the performance of predicting glioma patient survival after tumor resection.

## Data Availability

The raw data supporting the conclusions of this article will be made available by the authors, without undue reservation.
