# Supplementary material for: Practice of the new supervised machine learning predictive analytics for glioma patient survival after tumor resection: Experiences in a high-volume Chinese center
Source: Front Surg. 2023 Feb 17;9:975022. doi: 10.3389/fsurg.2022.975022 (PMC9981970; doi:10.3389/fsurg.2022.975022)
Supplement: Supplementary file 1 [file Datasheet1.zip › Supplementary Figure caption.docx]

Supplementary Figure 1. The flow chart of model construction.

Supplementary Figure 2. The calibration curve of Tree Gradient Boosting survival model in the prediction of 6-months, 12-months, 36-months and 60-months survival. A, prediction of 6-months survival; B, prediction of 12-months; C, prediction of 36-months; D, prediction of 60-months survival.

Supplementary Figure 3. The calibration curve of Component Gradient Boosting survival model in the prediction of 6-months, 12-months, 36-months and 60-months survival. A, prediction of 6-months survival; B, prediction of 12-months; C, prediction of 36-months; D, prediction of 60-months survival.

Supplementary Figure 4. The Forestplot of significant variables in the Cox proportional hazards model. The grey area at the plot referred to dummy variables of tumor subtype, with anaplastic oligoastrocytoma (NOS) as reference. (HR: hazards ratio; DA: diffuse astrocytoma; AA: anaplastic astrocytoma; GB: glioblastoma; OD: oligodendroglioma; AO: anaplastic oligodendroglioma; OA: oligoastrocytoma)
