# Supplementary material for: Practice of the new supervised machine learning predictive analytics for glioma patient survival after tumor resection: Experiences in a high-volume Chinese center
Source: Front Surg. 2023 Feb 17;9:975022. doi: 10.3389/fsurg.2022.975022 (PMC9981970; doi:10.3389/fsurg.2022.975022)
Supplement: Supplementary file 1 [file Datasheet1.zip › Supplementary Table 2.docx]

Supplementary Table2 The AUC and 95% CI of Tree Gradient Boosting Model

|  | AUC value | Lower limit of CI | Upper limit of CI |
| --- | --- | --- | --- |
| 6-months survival | 0.914 | 0.874 | 0.950 |
| 12-months survival | 0.863 | 0.832 | 0.905 |
| 36-months survival | 0.834 | 0.784 | 0.896 |
| 60-months survival | 0.845 | 0.787 | 0.888 |
