# Supplementary material for: Practice of the new supervised machine learning predictive analytics for glioma patient survival after tumor resection: Experiences in a high-volume Chinese center
Source: Front Surg. 2023 Feb 17;9:975022. doi: 10.3389/fsurg.2022.975022 (PMC9981970; doi:10.3389/fsurg.2022.975022)
Supplement: Supplementary file 1 [file Datasheet1.zip › Supplementary Table 3.docx]

Supplementary Table3 The AUC and 95% CI of Component Gradient Boosting Model

|  | AUC value | Lower limit of CI | Upper limit of CI |
| --- | --- | --- | --- |
| 6-months survival | 0.831 | 0.767 | 0.879 |
| 12-months survival | 0.851 | 0.792 | 0.879 |
| 36-months survival | 0.817 | 0.779 | 0.862 |
| 60-months survival | 0.826 | 0.772 | 0.881 |
