# Supplementary material for: Practice of the new supervised machine learning predictive analytics for glioma patient survival after tumor resection: Experiences in a high-volume Chinese center
Source: Front Surg. 2023 Feb 17;9:975022. doi: 10.3389/fsurg.2022.975022 (PMC9981970; doi:10.3389/fsurg.2022.975022)
Supplement: Supplementary file 1 [file Datasheet1.zip › Supplementary Table 4.docx]

Supplementary Table4 Sensitivity and specificity of Tree Gradient Boosting Model

|  | Sensitivity | Specificity |
| --- | --- | --- |
| 6-months survival | 94.4% | 91.3% |
| 12-months survival | 90.6% | 85.7% |
| 36-months survival | 99.3% | 71.3% |
| 60-months survival | 100% | 73.8% |
