# Supplementary material for: Practice of the new supervised machine learning predictive analytics for glioma patient survival after tumor resection: Experiences in a high-volume Chinese center
Source: Front Surg. 2023 Feb 17;9:975022. doi: 10.3389/fsurg.2022.975022 (PMC9981970; doi:10.3389/fsurg.2022.975022)
Supplement: Supplementary file 1 [file Datasheet1.zip › Supplementary Table 5.docx]

Supplementary Table5 Sensitivity and specificity of Component Gradient Boosting Model

|  | Sensitivity | Specificity |
| --- | --- | --- |
| 6-months survival | 90.0% | 84.1% |
| 12-months survival | 73.5% | 92.9% |
| 36-months survival | 85.2% | 87.5% |
| 60-months survival | 90.4% | 82.8% |
