# Supplementary material for: Practice of the new supervised machine learning predictive analytics for glioma patient survival after tumor resection: Experiences in a high-volume Chinese center
Source: Front Surg. 2023 Feb 17;9:975022. doi: 10.3389/fsurg.2022.975022 (PMC9981970; doi:10.3389/fsurg.2022.975022)
Supplement: Supplementary file 1 [file Datasheet1.zip › Supplementary Table 6.docx]

Supplementary Table6 Sensitive analysis of concordance Index of Models

|  | Tree Gradient Boosting Model | Component  Gradient  Boosting Model |
| --- | --- | --- |
| Diffuse astrocytoma | 0.869 | 0.823 |
| Oligodendroglioma | 0.874 | 0.812 |
| Glioblastoma | 0.837 | 0.792 |
