# Supplementary material for: Practice of the new supervised machine learning predictive analytics for glioma patient survival after tumor resection: Experiences in a high-volume Chinese center
Source: Front Surg. 2023 Feb 17;9:975022. doi: 10.3389/fsurg.2022.975022 (PMC9981970; doi:10.3389/fsurg.2022.975022)
Supplement: Supplementary file 1 [file Datasheet1.zip › Supplementary Table1.docx]

Supplementary Table 1. Module, Class and Hyperparameters in Python for Each Model

| Algorithm | Module and class | Hyperparameters for grid search | Hyperparameters selected |
| --- | --- | --- | --- |
| Cox proportional hazards  model | sksurv.linear_model.  CoxPHSurvivalAnalys  is |  | alpha = 0，ties ='breslow'，n_iter = 100，tol = 1e-09，verbose = 0 |
| Support Vector Machine model | sksurv.svm.FastSurvivalSVM | alpha:  [-12, -10, -8, -6,……,6, 8, 10, 12] | alpha=0,optimizer="rbtree", max_iter=1000,tol=1e-6, random_state=0 |
| Random Survival Forest  model | sksurv.ensemble.Ran  domSurvivalForest | n_estimators:  [10,20,30,……,80,90,100,200,400,600,……,4600,4800,5000],  min_samples_leaf: [3,4,5,6,……,18,19,20] | n_estimators=3000, max_depth=None, min_samples_split=6, min_samples_leaf=3, min_weight_fraction_leaf=0.0, max_features='auto', max_leaf_nodes=None, bootstrap=True, oob_score=False, n_jobs=4, random_state=None, verbose=0, warm_start=False, max_samples=None |
| Tree Gradient Boosting  Survival Model | sksurv.ensemble.Gra  dientBoostingSurvival  Analysis | n_estimators:  [10,20,30,……,80,90,100,200,300,400,……,2800,2900,3000],  learning_rate:  [0.01,0.1,0.2,0.3,0.4,0.5] | loss='coxph', learning_rate=0.01, n_estimators=2500, criterion='friedman_mse', min_samples_split=2, min_samples_leaf=1, min_weight_fraction_leaf=0.0, max_depth=3, min_impurity_split=None, min_impurity_decrease=0.0, random_state=None, max_features=None, max_leaf_nodes=None, presort='deprecated', subsample=1.0, dropout_rate=0.0, verbose=0, ccp_alpha=0.0 |
| Component Gradient  Boosting Survival Model | sksurv.ensemble.Co  mponentwiseGradient  BoostingSurvivalAnal  ysis | n_estimators:  [10,20,30,……,80,90,100,200,300,400,……,2800,2900,3000],  learning_rate:  [0.01,0.1,0.2,0.3,0.4,0.5] | loss='coxph', learning_rate=0.1, n_estimators=2900, subsample=1.0, dropout_rate=0, random_state=None, verbose=0 |
