# Supplementary figures and images for: Practice of the new supervised machine learning predictive analytics for glioma patient survival after tumor resection: Experiences in a high-volume Chinese center
Source: Front Surg. 2023 Feb 17;9:975022. doi: 10.3389/fsurg.2022.975022 (PMC9981970; doi:10.3389/fsurg.2022.975022)

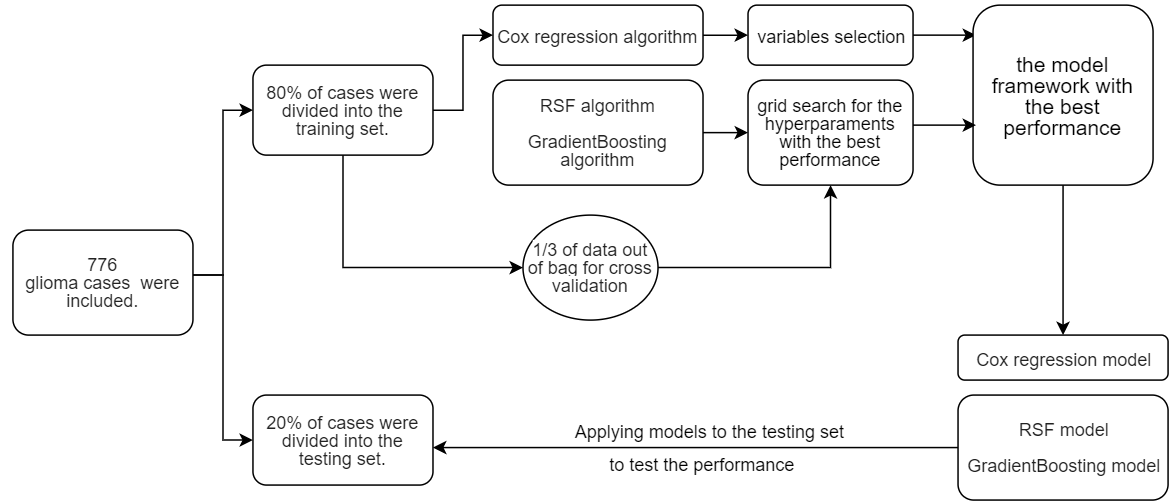

Supplement: Supplementary file 1 [file Datasheet1.zip › Supplementary Figure 1.png]

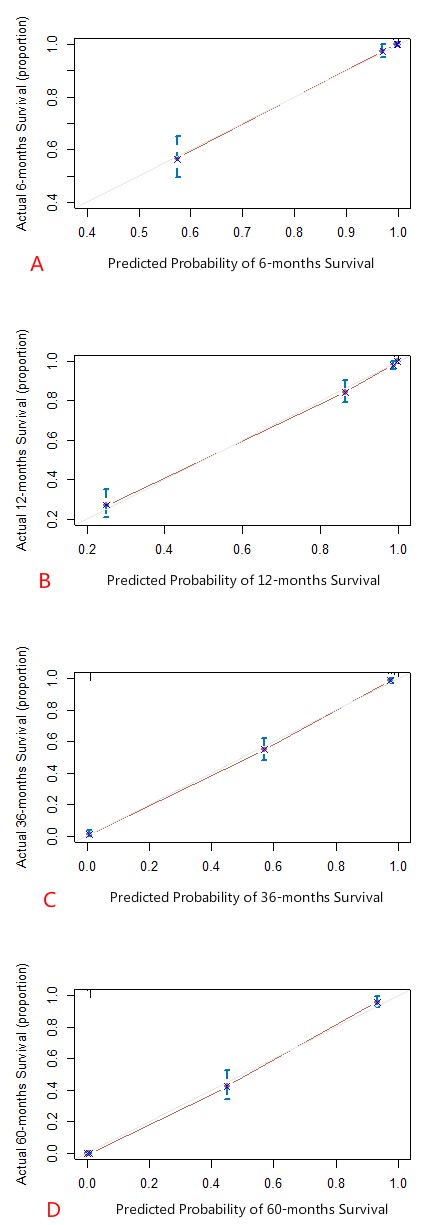

Supplement: Supplementary file 1 [file Datasheet1.zip › Supplementary Figure 2.jpg]

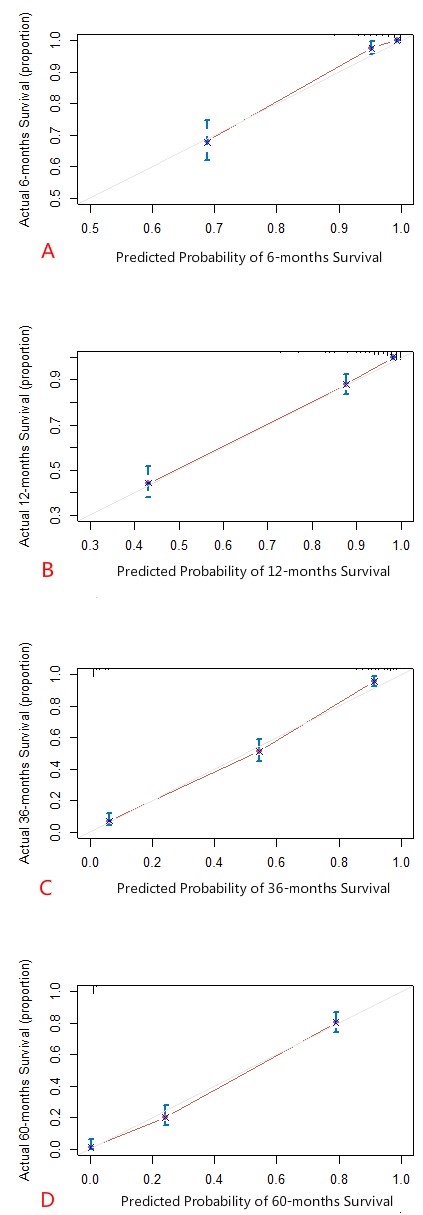

Supplement: Supplementary file 1 [file Datasheet1.zip › Supplementary Figure 3.jpg]

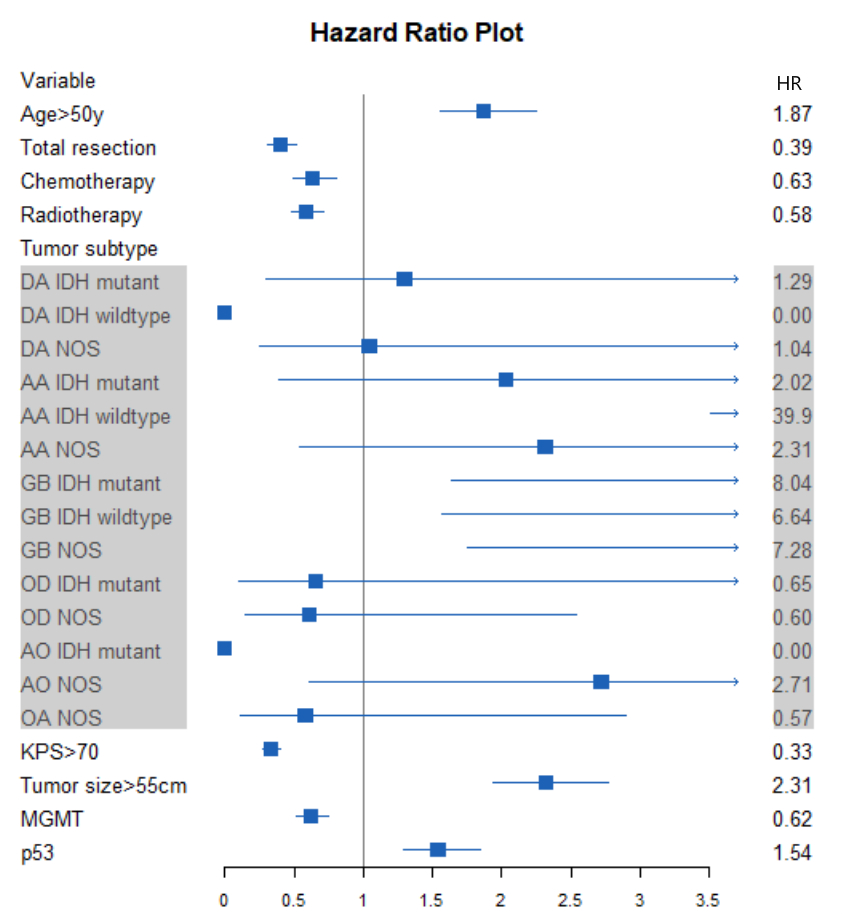

Supplement: Supplementary file 1 [file Datasheet1.zip › Supplementary Figure 4.jpg]
